# Medicine-food homology substances in cardiovascular disease prevention and management: from mechanisms to clinical evidence and future translation

**DOI:** 10.3389/fnut.2026.1825606

**Published:** 2026-06-25

**Authors:** Sifan Fei, Yanchen Rao, Jingxue Lai, Bing Zhang, Honggang Ma, Xueying Ma, Ying Liu

**Affiliations:** 1 Huzhou Central Hospital, Affiliated Central Hospital of Huzhou University, Huzhou, Zhejiang, China; 2 School of Medicine & Nursing, Huzhou University, Huzhou, Zhejiang, China; 3 Department of Neurology, The Third Affiliated Hospital of Sun Yat-Sen University, Guangzhou, Guangdong, China

**Keywords:** cardiovascular diseases, gut-heart axis, integrative medicine, medicine-food homology, multi-target mechanisms

## Abstract

Cardiovascular disease (CVD) is the leading global cause of death. “Medicine-food homology” (MFH), a fundamental principle of traditional Chinese medicine (TCM), emphasizes dual nutritional and therapeutic roles of natural substances, thereby attracting increasing attention in disease management. These substances exhibit synergistic effects through multiple targets and signaling pathways, demonstrating significant potential in the prevention, adjuvant therapy, and rehabilitation of CVD. This review presents the mechanisms underlying MFH substances (such as astragalus, codonopsis, and hawthorn) in targeting key pathways related to CVD, and summarizes findings from clinical randomized controlled trials (RCT). This review summarizes MFH substances’ role in CVD management, focusing on four core mechanisms (vascular defense, myocardial protection, gut-heart axis regulation, risk factor control) that mediate their multi-targeted, multi-pathway effects (e.g., anti-inflammation via Astragalus, myocardial repair via Ginseng). Clinical evidence confirms MFH substances enhance CVD therapeutic efficacy as adjuvant therapies, with mild adverse reactions, while highlighting the need for further research to advance translational application. Future research should prioritize large-scale multi-center trials, multi-omics integration, and standardized quality control to advance MFH from empirical use to precision medicine, providing new natural product-based strategies for comprehensive CVD prevention and treatment.

## 1 Introduction

Cardiovascular diseases (CVDs) refer to a group of disorders involving the heart and blood vessels (1). According to the World Health Organization (WHO) report released in July 2025 (2), CVDs mainly encompass coronary heart disease, cerebrovascular disease, peripheral arterial disease, rheumatic heart disease, congenital heart disease, deep vein thrombosis, and pulmonary embolism. CVD remains the leading cause of death worldwide, posing a significant public health challenge. According to the Global Burden of Disease Study, the global disability-adjusted life years (DALYs) for CVD reached 437 million (95% UI: 401 million to 465 million) in 2023, representing a 1.4-fold increase from 320 million (292 million to 344 million) in 1990 (3). Current mainstream treatment strategies include statins, antiplatelet therapy, and interventional procedures such as coronary artery bypass grafting and percutaneous coronary intervention. However, these interventions are associated with poor response, adverse effects, and surgery-related risks such as infection and cardiac rupture (4). Consequently, the diagnostic and therapeutic model is gradually shifting toward a prevention-centered approach (5), and accumulating evidence has demonstrated that improved dietary patterns and adherence to healthy lifestyles can effectively reduce the risk of CVD (6–8).

Among various prevention strategies, the theory of Medicine and Food Homology (MFH) has garnered increasing attention. “Medicine and Food Homology” refers to a class of natural substances with both nutritional and pharmacological properties. Originating from traditional Chinese medicine (TCM), the MFH concept emphasizes preventive healthcare and health maintenance through dietary regulation and system balance, consistent with the TCM principle of “treating disease before its onset” (9, 10). According to the “Administrative Provisions on the Catalogue of Substances: That Are Traditionally Both Food and Chinese Medicinal Materials” (2021) issued by the National Health Commission of China, a substance must meet two criteria to be recognized as an MFH substance: it must be traditionally used as food and must be listed in the Chinese Pharmacopoeia. As of 2024, China has officially listed 106 such substances, with common examples including goji berries, Chinese yam, red dates, and hawthorn. Unlike dietary supplements regulated by the U.S. FDA (which are mostly taken separately in the form of tablets or capsules) and traditional herbal medicinal products regulated by the European Medicines Agency (which are primarily registered and used as medicines), MFH substances in China possess a legally recognized dual identity as both food and medicine. These substances are widely used in modern functional foods, health products, and daily diets, emphasizing the achievement of disease prevention and health maintenance through diet, and serving as an important practical bridge connecting daily nutrition with traditional Chinese medicine theory (11–13) .

From the perspective of traditional medicine, CVD is not an isolated organ lesion but a local manifestation of systemic dysfunction. Multiple factors—such as chronic stress, emotional disturbances, irregular diet, overexertion, aging, and declining physical condition—can disrupt the body’s internal balance, thereby affecting blood circulation and organ function (14). In treating such conditions, TCM focuses on a holistic approach, alleviating symptoms by restoring systemic balance. The MFH theory is deeply rooted in this system. Based on their chemical composition and pharmacological characteristics, MFH substances can be classified into different categories, such as flavonoids, saponins, polysaccharides, and phenolic compounds, which will be discussed separately in the following mechanistic section. Both individual MFH-derived active components and compound formulations containing MFH-derived substances are discussed. Its material basis is not only rich in nutrients such as vitamins and minerals but also contains various natural bioactive components (e.g., flavonoids, saponins, and polysaccharides), conferring physiological functions including immune enhancement, fatigue relief, and sleep improvement. From a modern scientific perspective, the essence of MFH lies in the chemical similarity between many natural foods and medicines. Specific active components in foods can provide nutritional support and act on specific physiological targets, regulating metabolic pathways or molecular mechanisms to exert pharmacological activities (13). In clinical practice, the MFH concept has been applied to prevent and adjunctively treat various diseases, including cardiovascular and metabolic diseases. For example, hawthorn has been used for centuries as both a functional food and a medicinal plant, and studies have confirmed that it can regulate atherosclerosis progression by modulating cell survival and proliferation and inhibiting inflammatory responses (15). In another example, turmeric can be used as a daily cooking spice, and its active component curcumin is considered a potential dietary pancreatic lipase inhibitor for obesity prevention and management (16). MFH substances emphasize synergy between medicinal and dietary therapies, with active components acting simultaneously through multiple pathways and targets. For instance, active components such as adenosine, nonanoic acid, lauric acid, caprylic acid, and heptanoic acid derived from multiple medicinal herbs can act synergistically across pathways including lipid metabolism, atherosclerosis, and PI3K-Akt signaling, exerting antioxidant, anti-inflammatory, anti-platelet aggregation, and vasodilatory effects (17). Clinical studies have also suggested that adding Tongxinluo, a traditional Chinese medicine compound containing MFH-derived active components such as paeoniflorin and ginsenosides, to standard treatment provides significant benefits, reducing the incidence of major adverse cardiovascular and cerebrovascular events at 30 days (3.4% vs. 5.2%) and 1 year (5.3% vs. 8.3%), while lowering the risk of cardiac death and reinfarction with only mild gastrointestinal adverse reactions. This provides high-quality clinical evidence for the integrative treatment of ST-segment elevation myocardial infarction (STEMI) with traditional Chinese and Western medicine, highlighting the potential advantages of MFH-based traditional Chinese medicine compounds in the adjunctive treatment of CVD (17).

**FIGURE 1:**
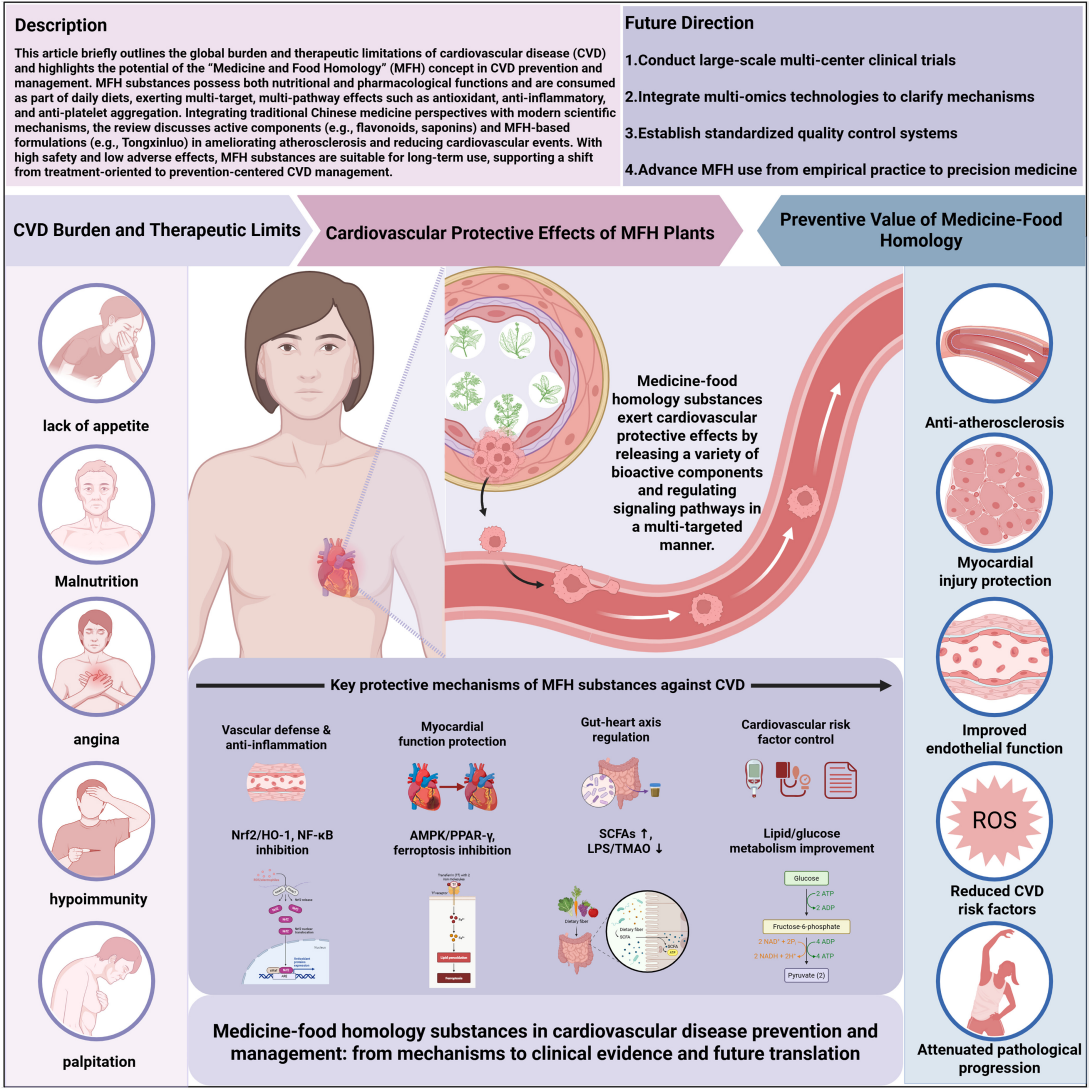
Medicine-food homology substances in cardiovascular disease prevention and management. This graphical abstract summarizes the role of medicine-food homology (MFH) substances in cardiovascular disease (CVD). It outlines the global burden of CVD, including symptoms such as anorexia, malnutrition, angina, hypoimmunity, and palpitations. The central panel illustrates the multi-target cardiovascular protective effects of MFH plants, which exert their actions by releasing a variety of bioactive components and regulating signaling pathways. Key protective mechanisms include vascular defense/anti-inflammation, myocardial function protection, gut-heart axis regulation, and cardiovascular risk factor control. On the right, the preventive values of MFH substances are listed, including anti-atherosclerosis, myocardial injury protection, improved endothelial function, reduced CVD risk factors, and attenuated pathological progression. Future directions for research are also proposed. (MFH, medicine-food homology; CVD, cardiovascular disease; ROS, reactive oxygen species; Nrf2, nuclear factor erythroid 2-related factor 2; HO-1, heme oxygenase-1; NF-κB, nuclear factor kappa B; AMPK, adenosine monophosphate-activated protein kinase; PPAR-γ, peroxisome proliferator-activated receptor gamma; SCFAs, short-chain fatty acids; LPS, lipopolysaccharide; TMAO, trimethylamine N-oxide). [Created in BioRender. Liu, Y. (2026) https://BioRender.com/5zh5bav].

In summary, MFH substances combine the safety of food with the functionality of medicine, with far fewer adverse effects than chemical drugs, making them particularly suitable for long-term use to enhance the body’s resistance (18). MFH corrects systemic imbalances through daily dietary regulation and provides personalized nourishment based on individual constitution and disease stage. This review elucidates the interaction between traditional MFH practices and modern medical approaches, confirming that such traditional methods still hold lasting practical value in contemporary healthcare systems. It is expected to generate new academic insights, further promote the translational research of MFH substances, and provide new ideas for exploring their potential application value in the management of CVD. Medicine-food homology substances show great potential for cardiovascular disease intervention, and their related functions and mechanisms are illustrated in Figure 1.

In the present study, we searched related publications from five major electronic databases (PubMed, Web of Science, Cochrane Library, CNKI, and Wanfang Data) between 2015 and 2026 while also incorporating landmark classic studies prior to this period. A set of standardized English and Chinese search terms were adopted, mainly covering the themes of medicine and food homology, traditional Chinese medicine, cardiovascular diseases, and related subtypes such as heart disease, cerebrovascular disease, peripheral arterial disease, rheumatic heart disease, congenital heart disease, deep vein thrombosis, and pulmonary embolism. Following the removal of duplicate records, a preliminary screening based on titles and abstracts was conducted, followed by a rigorous full-text evaluation of the remaining articles according to predefined inclusion and exclusion criteria. Finally, eligible original studies, clinical trials, mechanistic studies and relevant systematic reviews were included to support the thematic discussion, mechanism interpretation and clinical evidence summary of this review. In addition to monomeric substances listed in the pharmacopoeia, this review also includes compound preparations (e.g., capsules, tablets, and decoctions) in which MFH substances serve as the primary or principal constituent. The inclusion of compound formulations aligns with international classification standards for herbal medicinal products. Accordingly, the search strategy was supplemented with the term “Chinese Medicine” to capture relevant clinical evidence from compound formulations. The certainty of evidence for all included studies was assessed using the GRADE approach, with detailed ratings and justification provided in Supplementary material.

All representative MFH substances, bioactive components and related compound preparations involved in this review are summarized in Supplementary Table 2 in the Supplementary material.

## 2 Therapeutic mechanisms/effects of MFH substances on CVDs

**FIGURE 2:**
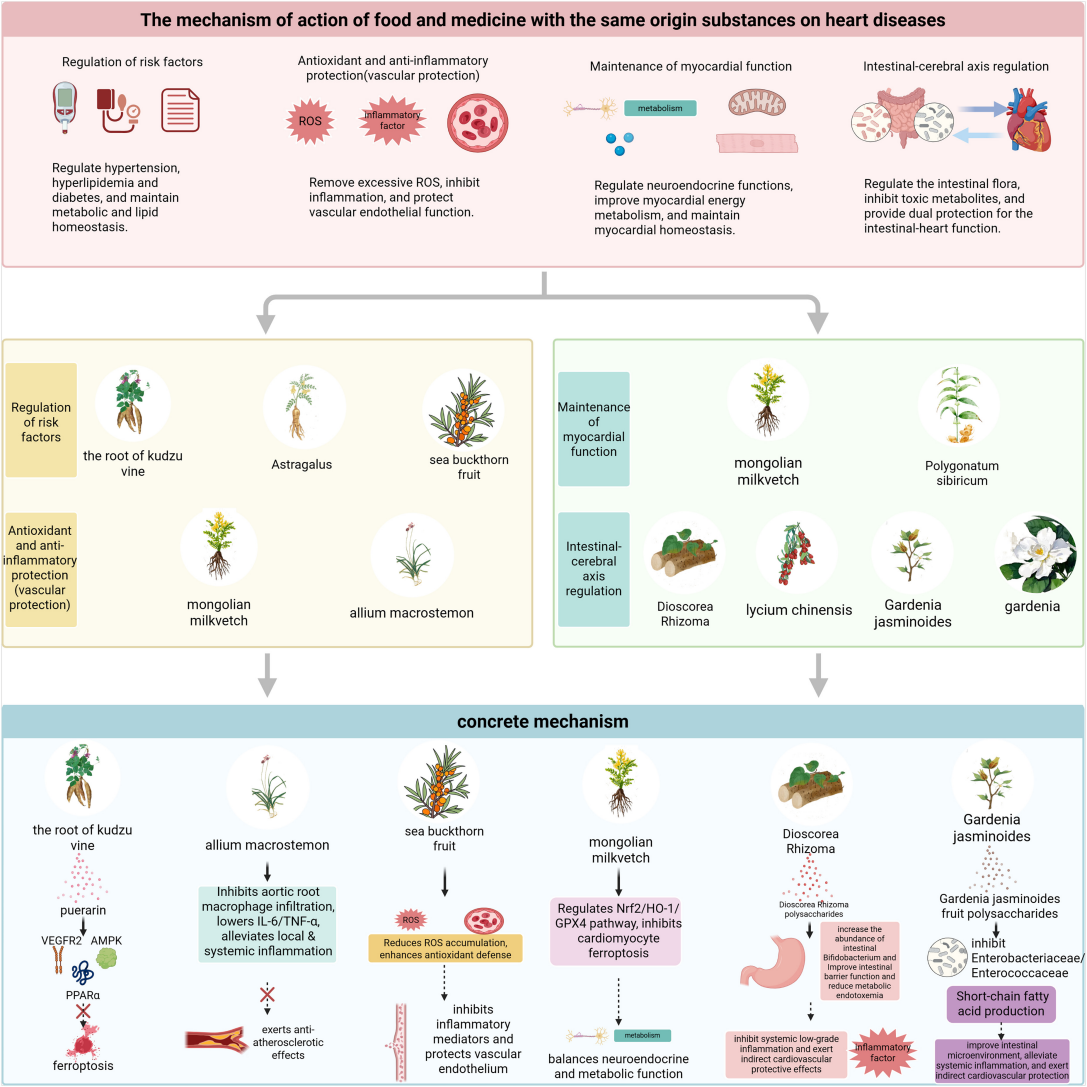
Mechanisms of action of medicine-food homology substances against cardiovascular diseases. This diagram illustrates the main mechanisms by which medicine-food homology (MFH) substances exert protective effects against heart diseases. The top panel categorizes these mechanisms into four main areas: regulation of risk factors, antioxidant and anti-inflammatory protection (vascular protection), maintenance of myocardial function, and intestinal-heart axis regulation. Representative MFH plants and their corresponding mechanisms are presented, including kudzu vine root, Astragalus, sea buckthorn fruit, Mongolian milkvetch, Allium macrostemon, Dioscorea Rhizoma, Lycium chinensis, Gardenia jasminoides, and Polygonatum sibiricum. The bottom panel details the concrete molecular mechanisms, such as the inhibition of ferroptosis by puerarin, reduction of ROS accumulation by sea buckthorn fruit, regulation of the Nrf2/HO-1/GPX4 pathway by Mongolian milkvetch, and production of short-chain fatty acids by Gardenia jasminoides fruit polysaccharides. Representative MFH substances were selected according to the official Catalogue of Medicine-Food Homology Substances issued by the National Health Commission of China and on the basis of available evidence for cardiovascular protection. These are illustrative examples and not an exhaustive list. (MFH, medicine-food homology; ROS, reactive oxygen species; Nrf2, nuclear factor erythroid 2-related factor 2; HO-1, heme oxygenase-1; GPX4, glutathione peroxidase 4; AMPK, adenosine monophosphate-activated protein kinase; VEGFR2, vascular endothelial growth factor receptor 2). [Created in BioRender. Liu, Y. (2026) https://BioRender.com/5zh5bav].

MFH substances exert cardiovascular protective effects via diverse pathways, and their specific action mechanisms are shown in Figure 2.

### 2.1 Protection against oxidative stress and inflammation (vascular defense)

Antioxidant and anti-inflammatory protections are crucial factors in the onset and progression of CVDs. In CVD, elements such as high glucose levels and oxidized low-density lipoproteins stimulate vascular endothelial cells and cardiomyocytes to produce excessive Reactive Oxygen Species (ROS), surpassing the clearance capacity of the endogenous antioxidant system, which includes enzymes like superoxide dismutase (SOD) and glutathione peroxidase (GSH-Px). This overproduction of ROS leads to lipid peroxidation, impairs vascular endothelial function by inhibiting endothelial nitric oxide synthase (eNOS) activity and reducing nitric oxide (NO) production, and promotes the release of inflammatory factors, thereby accelerating pathological processes such as atherosclerosis and myocardial injury. Notably, as a key risk factor for CVD, psychosocial stress can also induce specific inflammatory responses, forming a “stress-inflammation-CVD” pathological chain (19). Active components derived from various natural ingredients offer significant protective effects for the cardiovascular system by precisely modulating oxidative pathways and inhibiting inflammation and its cascading reactions. For instance, the hydrophilic extract of Salvia miltiorrhiza (SMHE) targets markers such as malondialdehyde (MDA), glutathione (GSH), SOD, and glutathione reductase (GSSG-R) to regulate endogenous antioxidant pathways. In diabetic patients with CHD, a 30-day treatment with SMHE significantly decreased serum levels of the oxidative stress marker MDA (20). In human knee osteoarthritis, degenerated chondrocytes exhibit a significant reduction in the protein expression of nuclear factor kappaB (NF-κB), Interleukin (IL)-6, and tumour necrosis factor alpha (TNF-α) when treated with astragaloside IV, thereby inhibiting the inflammatory response (21). The nuclear factor erythroid 2-related factor 2 (Nrf2)/heme oxygenase-1 (HO-1) pathway is recognized as a crucial signaling pathway for antioxidant defense. Astragalus polysaccharide (ASTP), which targets HO-1, Nrf2, and kruppel-like factor 2 (KLF2), exerts protective effects by promoting the Nrf2/HO-1 and Mitogen-activated protein kinase (MEK)/extracellular regulated protein kinases (ERK) pathways, effectively safeguarding vascular endothelial cells from oxidative damage (22). Saponins derived from Allii Macrostemonis Bulbus (SAMB) target macrophages in the aortic root and demonstrate anti-atherosclerotic effects by inhibiting macrophage infiltration, while significantly reducing serum levels of inflammation-related factors such as IL-6 and TNF-α (with Interleukin-1β (IL-1β) levels showing no significant change), thereby alleviating both local and systemic inflammatory responses (23). Additionally, secondary metabolites derived from medicinal and food homologous plants exhibit anti-inflammatory properties by inhibiting the production of inflammatory mediators and cytokines, regulating NF-κB and mitogen-activated protein kinases (MAPK) pathways, and modulating antioxidant-related pathways, ultimately improving symptoms of various inflammation-related diseases. The consumption of these medicinal and edible homologous plants as food may enhance immune system function (24). This not only enriches the theoretical understanding of the role of natural components in preventing CVDs but also serves as a significant reference for the future development of dietary supplements or adjunct treatment plans that integrate antioxidant and anti-inflammatory effects.

### 2.2 Preservation of myocardial function

The preservation of myocardial function can be achieved through two interrelated mechanisms: neuroendocrine and metabolic regulation, as well as the enhancement of myocardial energy metabolism and functional performance. These mechanisms act synergistically to establish a comprehensive cardiovascular protective system.

In the pathological state of CVDs, the neuroendocrine system—including the sympathetic nervous system and the renin-angiotensin-aldosterone system (RAAS)—becomes abnormally overactivated. This overactivation further increases cardiac workload, induces vascular endothelial injury, and exacerbates myocardial structural remodeling (25). Concurrently, metabolic disturbances such as glycolipid metabolism disorders and mitochondrial energy metabolism dysfunction frequently occur in cardiomyocytes. Neuroendocrine hyperactivity and metabolic abnormalities mutually reinforce each other, forming a vicious pathological cycle that accelerates the deterioration of cardiac function and the progression of CVDs (26). MFH components regulate myocardial energy and glycolipid metabolism through dual pathways. Small-molecule active substances are absorbed into the bloodstream and target key signaling axes such as AMPK/SIRT1, Nrf2, and NF-κB, thereby improving glucose and lipid utilization and maintaining myocardial energy homeostasis. In contrast, poorly absorbed components such as polysaccharides modulate the gut microbiota. They promote beneficial metabolite production and alleviate insulin resistance and systemic inflammation via the gut-metabolism-heart axis, indirectly ameliorating myocardial glycolipid disorders (27). Current clinical pharmacological interventions primarily target these core pathological links to delay adverse ventricular remodeling and improve prognosis.

Bioactive components derived from Leguminosae MFH Chinese medicines—including saponins (e.g., astragaloside IV, soyasaponin I) and flavonoids (e.g., puerarin, calycosin, isoliquiritigenin, genistein) from Astragalus membranaceus, Pueraria lobata, Glycyrrhiza uralensis, and Glycine max—exert multi-target protective effects on both cardiovascular and nervous system injuries. In the cardiovascular system, the overactivation of neuroendocrine systems (RAAS and sympathetic nerve), along with glycolipid metabolic disorders and mitochondrial dysfunction, interact and reinforce one another, forming a vicious cycle that accelerates the progression of myocardial hypertrophy, fibrosis, and heart failure (28). These herbal ingredients regulate signaling pathways such as Nrf2/HO-1, NF-κB/MAPK, and PI3K/Akt/mTOR, thereby inhibiting oxidative stress and inflammatory responses, reducing cardiomyocyte apoptosis, improving mitochondrial energy metabolism, and ultimately delaying cardiac remodeling. In neurological diseases—including Alzheimer’s disease, Parkinson’s disease, and stroke—they also exert neuroprotective effects by reducing ROS production, suppressing neuroinflammation, inhibiting the mitochondrial apoptosis pathway (Bax/Bcl-2/caspase-3), and restoring mitochondrial membrane potential (28). Thus, Leguminosae MFH Chinese medicines hold the potential to regulate the common pathological axis of oxidation-inflammation-mitochondria-apoptosis, providing a theoretical basis for the combined intervention of cardiovascular and cerebrovascular diseases (26, 28). Bioactive components derived from medicinal and edible homologous plants—such as mulberry leaf polysaccharide, astragalus polysaccharide, Siraitia grosvenorii polysaccharide, and angelica polysaccharide—can regulate glycolipid metabolism and insulin resistance through multiple pathways (29). Specifically, mulberry leaf polysaccharide repairs insulin signaling by inhibiting PTP1B and activating the PI3K-AKT pathway; astragalus polysaccharide activates AMPK signaling to promote glucose uptake and improve insulin sensitivity; Siraitia grosvenorii polysaccharide suppresses the TLR4-NF-κB inflammatory pathway and enhances antioxidant capacity; and angelica polysaccharide modulates macrophage polarization and inhibits the secretion of inflammatory factors via activation of the Nrf2/HO-1 pathway (29). Collectively, these medicinal and edible homologous polysaccharides improve pancreatic islet function, correct glycolipid metabolic disorders, alleviate oxidative and inflammatory damage, and maintain energy metabolism homeostasis by regulating key signaling pathways including PI3K-AKT, AMPK, NF-κB, and Nrf2/Keap1 (29). In terms of neuroendocrine and metabolic regulation, bioactive components in Astragalus target iron transport-related proteins as well as Nrf2, HO-1, and glutathione peroxidase 4 (GPX4), thereby inhibiting ferroptosis through modulation of the Nrf2/HO-1/GPX4 pathway (30). Natural active ingredients target key nodes of neuroendocrine regulation, balancing autonomic nerve function and neurotransmitter metabolism to stabilize heart rhythm and blood pressure. They also intervene in the RAAS system to correct metabolic abnormalities caused by hormonal disorders, thereby constructing a cardiovascular protection network characterized by synergistic regulation between neuroendocrine and metabolic processes.

Disordered myocardial energy metabolism and functional impairment are core factors in the progression of CVDs (31). Various active components from natural ingredients provide significant support by regulating energy metabolism pathways and maintaining myocardial structural stability. For instance, polysaccharides and steroidal saponins derived from Polygonatum sibiricum (Huangjing, a documented MFH plant) target AMPK and PPAR-γ as key pathways to improve cellular energy metabolism and glucose-lipid homeostasis (31–33). These components activate AMPK to promote glycolysis and ATP generation, enhance insulin sensitivity, and upregulate the expression of GLUT4 and glucokinase. Meanwhile, their modulation of the PPAR-γ coactivator-1α pathway improves mitochondrial function and reduces oxidative stress. Concurrently, they exert anti-inflammatory effects by suppressing NF-κB and TLR4 signaling, thereby attenuating systemic low-grade inflammation and metabolic endotoxemia (34). This integrated metabolic and anti-inflammatory modulation can ameliorate dyslipidemia, obesity-induced insulin resistance, and atherosclerosis, offering significant protective potential against CVDs driven by metabolic dysfunction (34). In summary, natural active ingredients form a protective network against the vicious cycle of CVDs by synergistically regulating neuroendocrine and metabolic functions while improving myocardial energy metabolism.

### 2.3 Regulation of gut-heart axis

The “gut-heart axis” refers to the bidirectional regulatory network between the gut and the heart, mediated by microbial metabolism, immune regulation, inflammatory signals, and neuroendocrine communication. In a healthy state, the gut microbiota maintains a dynamic balance: beneficial bacteria form a biological barrier through intestinal mucosal colonization, reducing endotoxin production; at the same time, they metabolize dietary fiber to generate short-chain fatty acids (SCFAs) and other active substances, which act on cardiovascular tissues via the circulatory system, exerting protective functions such as regulating vascular tone, inhibiting inflammatory responses, and improving myocardial energy metabolism. Conversely, gut microbial dysbiosis is a major risk factor for the onset and progression of CVDs. When SCFA-producing beneficial bacteria decrease and harmful *Enterobacteriaceae* increase, excessive amounts of toxic metabolites such as trimethylamine N-oxide (TMAO) and lipopolysaccharide (LPS) are produced. These substances enter the bloodstream, damage vascular endothelial cells by inhibiting nitric oxide synthesis and activating inflammatory pathways, promote atherosclerotic plaque formation, and may induce hypertension, heart failure, and coronary artery disease. To address this issue, dietary adjustments (e.g., high-fiber diets), probiotic or prebiotic supplementation, and reduced intake of red meat and high-fat foods can effectively modulate gut microbiota composition and reduce CVD risk (35). Further studies indicate that SCFAs act primarily through two pathways: first, by activating G-protein-coupled receptors (GPR41/GPR43), thereby regulating vascular tone and lowering blood pressure; and second, by inhibiting histone deacetylases, which reduces the release of inflammatory cytokines and facilitates the repair of myocardial mitochondrial function, thus delaying CVD progression (36, 37). Notably, probiotics can indirectly affect CVDs by improving blood pressure and mood through gut microbiota regulation, which aligns with the core mechanism of the gut-heart axis. Probiotics enrich beneficial microbial populations, enhance SCFA production, and mitigate inflammatory responses, thereby contributing to cardiovascular homeostasis (35).

Accumulating evidence suggests a bidirectional regulatory relationship between MFH substances and the gut microbiota. On one hand, the gut microbiota metabolizes natural active components from MFH substances into small-molecule compounds that can be utilized by the host; Moreover, MFH substances help maintain intestinal microenvironment homeostasis and protect host health by modulating the composition and metabolic activity of the gut microbiota, while the gut microbiota, in turn, influences the pharmacological efficacy of these active components through their biotransformation *in vivo* (38). This mutual interaction establishes a positive feedback loop, opening new avenues for disease prevention and adjunctive intervention.

Based on this theoretical framework, recent studies have increasingly focused on the gut microbiota-regulatory effects of specific MFH plants and their associated cardiovascular benefits. MFH plants have garnered attention due to their favorable safety profiles and diverse bioactivities. Research indicates that they holistically modulate gut microbiota composition and function in a context-dependent manner, promoting a resilient ecological balance rather than targeting specific “beneficial” or “harmful” bacteria in isolation. This regulation exerts therapeutic effects through multi-pathway interactions along the microbiota-metabolism-immune axis (39).

Take Pueraria montana var. thomsonii as an example. This plant, valued for both medicinal and edible purposes, contains a low-molecular-weight polysaccharide (RPP-2) in its roots that regulates gut microbiota, notably affecting the abundance of the genus *Oscillibacter*. The Framingham Heart Study first revealed that *Oscillibacter* possesses cholesterol-degrading capabilities (including glycosylation and dehydrogenation), effectively reducing cholesterol levels in feces and plasma. This finding provides a mechanistic basis for understanding how RPP-2 exerts its cholesterol-lowering and cardiovascular protective effects by modulating intestinal microecology, particularly by promoting *Oscillibacter* proliferation, further highlighting the multifaceted health value of Pueraria montana var. thomsonii within the MFH framework (40, 41).

Other MFH-derived active components have also been shown to confer cardiovascular protection via gut microbiota modulation. For instance, Lycium barbarum polysaccharide enhances the abundance of SCFA-producing bacteria in the gut of type 2 diabetic mice, promotes acetate and propionate synthesis, activates the GPR41/GPR43 pathway to improve insulin sensitivity, and reduces pro-inflammatory bacteria and LPS release. Ginkgolide B effectively modulates the gut microbiota in high-fat diet-induced ApoE^–^/^–^ mice and inhibits hepatic flavin-containing monooxygenase 3 (FMO3) expression, thereby reducing the conversion of trimethylamine (TMA) to TMAO. This results in a 45%–50% reduction in serum TMAO levels and a 25%–30% decrease in atherosclerotic plaque area (38).

Beyond single-component studies, the review by Xia and Xiao (42) systematically summarized the gut microbiota-regulating functions of various MFH substances. For example, water extract of Lycium barbarum L. leaves significantly modulated gut microbiota composition in a type 2 diabetic rat model, reducing the abundance of genera such as *Parasutterella* and *Blautia*, reversing the *Firmicutes*/*Bacteroidetes* ratio, and simultaneously ameliorating liver, kidney, and pancreatic damage. Furthermore, a preliminary study in patients with type 2 diabetes showed that consumption of yam (Dioscorea Rhizoma) porridge increased the abundance of beneficial bacteria such as *Bifidobacterium adolescentis* and *Bifidobacterium infantis* in the gut (42). These gut microbiota-modulating effects help maintain intestinal barrier function and reduce metabolic endotoxemia, thereby potentially exerting indirect cardiovascular protection through the inhibition of systemic low-grade inflammation.

Further mechanistic insights are provided by studies on raspberry polysaccharide (RP2), which elucidate the anti-obesity and cardiovascular protective mechanisms of MFH polysaccharides. RP2 significantly improves high-fat diet-induced obese phenotypes, including reduced weight gain, decreased fat accumulation, corrected dyslipidemia and glucose intolerance, and alleviated liver pathological damage and oxidative stress. As a prebiotic, RP2 modulates gut microbiota composition by increasing the abundance of SCFA-producing beneficial bacteria (e.g., *Lactobacillus* and *Bifidobacterium*), inhibiting pro-inflammatory bacteria (e.g., *Olsenella* and *Ruminiclostridium_9*), enhancing intestinal barrier function, upregulating tight junction proteins (Occludin, Claudin-5, ZO-1), and reducing endotoxin leakage into the bloodstream, thereby alleviating systemic low-grade inflammation. Additionally, RP2 inhibits TLR4/NF-κB signaling activation in the liver, reduces pro-inflammatory cytokine levels (TNF-α, IL-6, IL-1β), and attenuates oxidative stress. Collectively, these effects improve obesity-related metabolic disorders and offer potential protection against obesity-induced cardiovascular risk factors, including dyslipidemia, insulin resistance, and chronic inflammation (43) .

Active polysaccharides from other MFH sources similarly demonstrate potential in preventing and treating CVDs by regulating the gut microbiota-metabolism-immunity axis. Ganoderma lucidum polysaccharide, for example, reduces potentially harmful bacteria (e.g., *Clostridium_sensu_stricto_1*) while increasing SCFA-producing beneficial bacteria (e.g., *Prevotellaceae_UCG-001* and *Ruminococcaceae_UCG-009*), thereby promoting dietary fiber fermentation to generate more SCFAs and inhibiting the production of harmful metabolites such as LPS and TMAO. These changes improve host metabolic function and exert anti-inflammatory, lipid-lowering, and cardiovascular protective effects. Likewise, polysaccharides derived from the fruit of Gardenia jasminoides suppress potentially harmful bacteria (e.g., *Enterobacteriaceae* and *Enterococcaceae*) while promoting the proliferation of beneficial *Bacteroides*-like taxa. Through this microbiota remodeling, these polysaccharides increase SCFA production, maintain intestinal barrier integrity, and reduce inflammatory responses and bile acid levels, thereby improving the intestinal microenvironment and host metabolism.

Despite their diverse origins, MFH substances share common mechanisms of gut microbiota regulation—namely, increasing SCFA-producing bacteria, reducing harmful metabolites, and maintaining intestinal barrier function—that directly target key pathological processes in CVD development, including chronic low-grade inflammation, dyslipidemia, and vascular damage mediated by toxins such as TMAO. Consequently, MFH substances such as Ganoderma lucidum and Gardenia jasminoides fruit, by modulating the gut-heart axis, represent promising candidates for dietary intervention and adjunctive therapy for CVDs (38). In summary, bioactive components from MFH plants exert therapeutic effects through multi-axis crosstalk within the microbiota-metabolism-immunity network, not only mitigating common adverse effects associated with conventional pharmacotherapies (e.g., gut dysbiosis) but also targeting the fundamental pathophysiological origins of disease progression.

**FIGURE 3:**
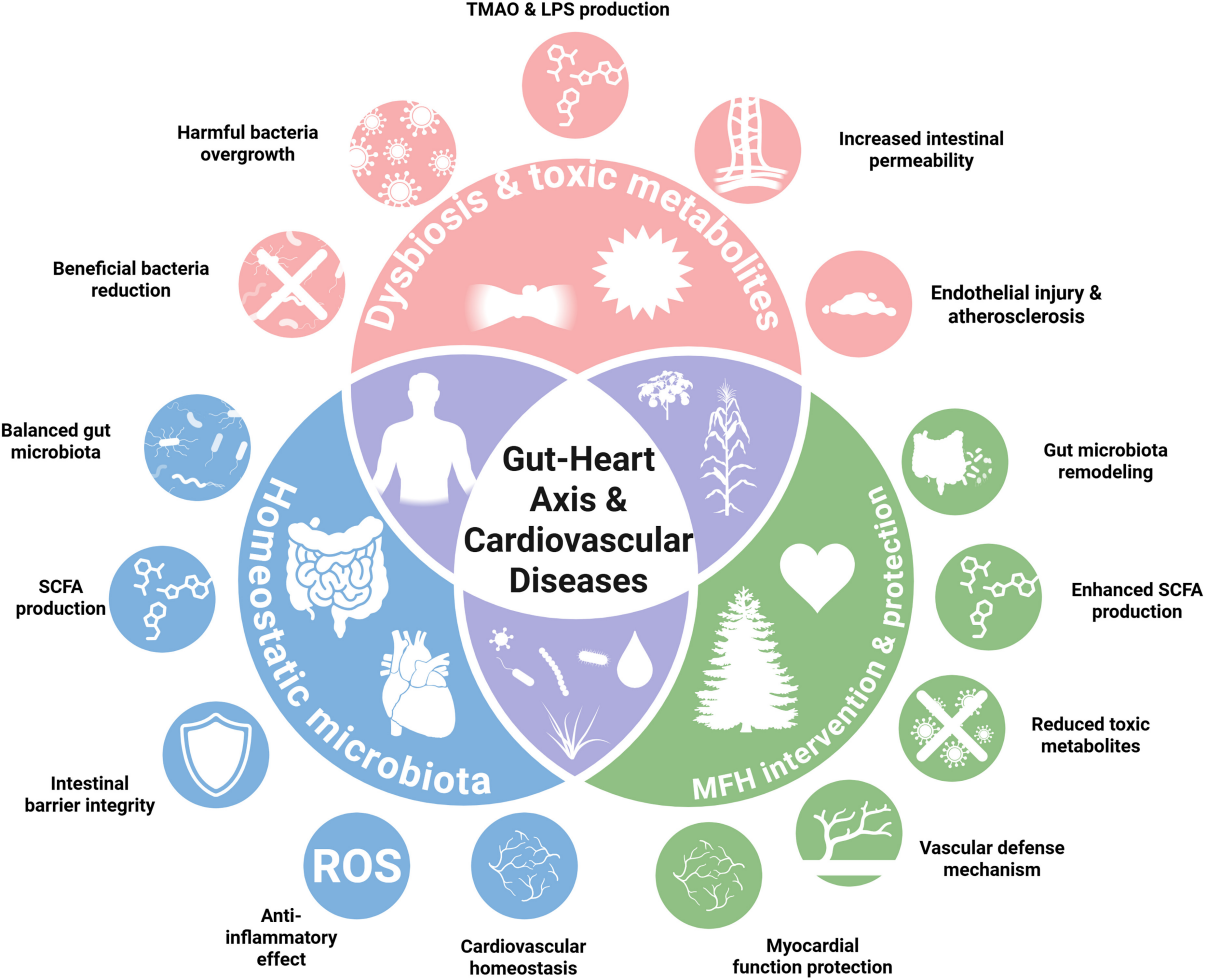
The gut-heart axis in cardiovascular diseases: homeostasis, dysbiosis, and intervention by medicine-food homology substances. This schematic illustrates the tripartite relationship between gut microbiota, the gut-heart axis, and cardiovascular diseases (CVD). The central theme is the “Gut-Heart Axis & Cardiovascular Diseases.” The blue sector depicts a state of homeostatic microbiota, characterized by balanced gut flora, short-chain fatty acid (SCFA) production, intestinal barrier integrity, anti-inflammatory effects, and cardiovascular homeostasis. The red sector shows pathological dysbiosis, including harmful bacteria overgrowth, beneficial bacteria reduction, production of toxic metabolites (TMAO & LPS), increased intestinal permeability, and subsequent endothelial injury and atherosclerosis. The green sector highlights the protective role of medicine-food homology (MFH) intervention, which remodels the gut microbiota, enhances SCFA production, reduces toxic metabolites, and provides vascular defense and myocardial function protection. (CVD, cardiovascular disease; MFH, medicine-food homology; SCFA, short-chain fatty acid; TMAO, trimethylamine N-oxide; LPS, lipopolysaccharide). [Created in BioRender. Liu, Y. (2026) https://BioRender.com/5zh5bav].

In summary, MFH substances bidirectionally interact with the gut microbiota to regulate the gut-heart axis. They are metabolized by gut microbes into active compounds, while also reshaping microbial composition to enhance SCFA production, reduce pro-inflammtory metabolites such as TMAO and LPS, and maintain intestinal barrier integrity. Examples include Pueraria polysaccharide promoting cholesterol-degrading *Oscillibacter*, Lycium polysaccharide activating GPR41/GPR43, and ginkgolide B lowering TMAO by 45–50%. Collectively, these findings suggest that MFH substances offer a promising adjunctive strategy for CVD prevention via microbiota- metabolism-immune modulation (44–48). Intestinal flora participates in CVD progression, and the gut-heart axis regulatory mechanisms mediated by MFH substances are displayed in Figure 3.

### 2.4 Comprehensive management of cardiovascular risk factors

The above-mentioned direct mechanisms of action (oxidative stress inhibition, myocardial protection, and gut-cardiac axis regulation) can also indirectly exert the preventive and therapeutic effects of CVD by synergistically regulating core risk factors such as hypertension and dyslipidemia. The multi-target regulatory characteristics are as follows: MFH substances play a crucial role in the primary prevention and integrated management of key cardiovascular risk factors, such as hypertension, dyslipidemia, and diabetes. This preventive function often precedes and complements their direct therapeutic effects, embodying the concept of “preventive treatment of disease” in traditional medicine. Rather than acting through isolated pathways, MFH ingredients commonly employ a multi-targeted strategy to restore metabolic homeostasis. For instance, they can simultaneously regulate lipid profiles, improve insulin sensitivity, and mitigate endothelial dysfunction by modulating interconnected signaling pathways related to metabolism and inflammation. This systemic approach helps reduce the overall burden of risk factors, thereby delaying the onset and progression of CVDs. The evidence presented in the preceding sections, including the regulation of oxidative stress, inflammation, and gut microbiota, collectively contributes to this risk-factor-modifying effect. Therefore, the value of MFH extends beyond adjunctive treatment to encompass a broader strategy for cardiovascular risk reduction and long-term health maintenance.

For example, curcuminoids from turmeric (Curcuma longa), an MFH spice widely used in daily cooking, act as potent reversible competitive inhibitors of pancreatic lipase (IC_50_ = 0.52 mg/mL for curcumin), thereby reducing dietary fat digestion and absorption (16). Screening of 20 MFH plants further identified hawthorn fruit, lotus leaf, and sea buckthorn fruit as additional significant lipase inhibitors (14), highlighting the diversity of MFH resources in metabolic intervention. By blocking dietary fat hydrolysis at the enzymatic level, these MFH components offer a preventive strategy against obesity-driven dyslipidemia and subsequent cardiovascular risk.

As summarized in Supplementary Table 1, diverse categories of MFH active substances (e.g., triterpenoid saponins, phenolic acids, and medicinal foods) exert cardioprotective effects through distinct bioactive components and targeted signaling pathways, covering oxidative stress alleviation, inflammation suppression, myocardial function preservation, gut-heart axis regulation, and cardiovascular risk factor management. These preclinical findings provide a solid mechanistic foundation for the subsequent discussion of clinical evidence and translational applications of MFH in CVD.

The cardioprotective effects of MFH substances are mediated through multiple coordinated mechanisms. These mechanisms mainly include cardiovascular risk factor regulation, antioxidant and anti-inflammatory protection, myocardial function maintenance, and gut-heart axis modulation, with each pathway involving specific MFH components and targeted signaling molecule.

## 3 Clinical basis for the prevention and treatment of heart disease with MFH

In clinical practice for CVDs, MFH substances are not merely alternatives to pharmaceuticals; rather, they function as adjuvant agents to enhance patient conditions and improve therapeutic efficacy through synergistic interactions with conventional treatments. Jin et al. employed rigorous scientific methods to screen a formula comprising seven MFH materials—Codonopsis pilosula, Astragalus membranaceus, Platycodon grandiflorum, Taoren, Glycyrrhiza, Danggui, and Allium macrostemon—from traditional prescriptions. They identified the primary effective components, action targets, and pathways of this formula, thereby substantiating its potential to ameliorate microvascular angina through mechanisms such as anti-inflammatory effects, vascular protection, and antiplatelet aggregation. This research provides a promising and easily acceptable new direction for the management of this challenging form of angina (17). Jalaly et al. demonstrated that in patients with stable angina pectoris, the addition of a 12-week course of standardized Crataegus oxyacantha extract (Cratagol, 240 mg tablets standardized to 4–6 mg vitexin-2″-O-rhamnoside per tablet, twice daily) to conventional therapy significantly reduces serum levels of intercellular adhesion molecule-1 (ICAM-1) and E-selectin (*P* < 0.01), indicating attenuated endothelial inflammation and leukocyte adhesion. Notably, the combination of hawthorn extract with aerobic exercise produced a synergistic anti-inflammatory effect superior to either intervention alone *(P* = 0.021), suggesting that MFH-based adjuvant therapy integrated with lifestyle modification may offer enhanced cardiovascular protection through anti-atherosclerotic mechanisms (49). In patients with acute STEMI receiving conventional pharmacological treatment, the adjunctive use of Tongxinluo Capsules has been shown to reduce major adverse cardiovascular events from 30 days to 1 year post-infarction, as well as to attenuate myocardial injury, while exhibiting manageable side effects (50). Furthermore, integrated treatment that combines TCM and Western medicine represents a promising approach for managing CVDs. Shen et al. (51) reported that such integrative strategies can enhance cardiac function in patients with heart failure, while maintaining a high level of safety (52). TCM may address certain unmet therapeutic needs not fully covered by Western medicine. Notably, improper integration of TCM and Western medicine may lead to counterproductive outcomes, potentially compromising the efficacy and safety of combined treatments for cardiovascular conditions (53). In the adjuvant treatment of heart disease, MFH substances adhere to the principle of “targeting disease mechanisms and synergizing with conventional treatments.” This approach not only addresses the limitations of single-drug therapy in terms of symptom relief and quality of life improvement but also minimizes the risk of requiring additional interventions due to their inherent natural properties. However, it is crucial to emphasize that their application should be conducted under the supervision of a physician or clinical nutritionist. The selection of MFH substances must be tailored to the patient’s specific condition, medication regimen, and individual physical constitution to effectively achieve the therapeutic objective of enhancing adjuvant efficacy while ensuring safety and minimal harm.

Building on the previous discussion regarding the preventive effects and adjuvant treatment progress of MFH substances for heart disease, a systematic summary of evidence-based medical research can further substantiate their clinical application, elucidating their practical value and applicable boundaries. This article presents this evidence-based medical evidence in a tabular format (Table 1). Notably, the preparations listed are TCM compounds containing MFH ingredients rather than pure MFH substances, given that rigorous RCT evidence for single-MFH-herb cardiovascular interventions remains limited. This article presents this evidence-based medical evidence in a tabular format and will not elaborate further here.

**TABLE 1:** Clinical evidence, efficacy and research limitations of MFH-related preparations in the treatment of CVDs.

Literature	Subclassification of diseases	Level of evidence	Research results	Research limitations	GRADE certainty of evidence
Qiliqiangxin (55)	Heart failure	RCT ChiCTR1900021929	Reduces the risk of heart failure hospitalization and cardiovascular death; alleviates the severity of heart failure; safe.	Limited applicability; unexplored drug synergy; treatment exclusion bias; disease specificity; follow-up affected	Moderate
Shensong Yangxin (56)	Atrial fibrillation	RCT ChiCTR registration: ChiCTR1900026912	For patients who have undergone catheter ablation for persistent atrial fibrillation, 12-month adjunctive treatment with Shensong Yangxin Capsules can effectively reduce the recurrence rate, improve the quality of life, and has good safety.	This study has limitations, including the potential underdetection of atrial fibrillation recurrence, non-significant secondary outcomes, incomplete follow-up in some patients, unclear optimal target population, insufficient quality-of-life assessment, and limited generalizability to other populations.	Low
Yangxin Recipe (84)	Coronary heart disease	RCT Registration Number: 2020SHL-KYYS-134	The combination of the heart-nourishing formula and conventional Western medicine has a significant synergistic effect, which can better relieve angina pectoris and improve symptoms such as coronary artery stenosis. Its therapeutic effect is related to the anti-atherosclerotic effects of the ingredients in the formula, such as Salvia miltiorrhiza and Astragalus membranaceus.	The study has limitations, including unexplored key indicators, unclear mechanisms of the Yangxin prescription, a limited population scope and follow-up duration, and unexcluded confounding factors.	Low
Danhong injection (85)	Anginapectoris due to coronary heart disease	OSF Registration number: DOI 10.17605/ OSF.IO/TPZJ5	This study evaluates the efficacy of Danhong Injection in improving cardiac function and regulating blood lipids in CHD patients with angina pectoris.	The follow-up was too short to evaluate long-term effects, and it remains unclear whether the injection can serve as an alternative to conventional treatment.	Low
Shuxuening injection (86)	Unstable angina pectoris	RCT ChiCTR registration number: ChiCTR1800019754	As an adjunct to conventional therapy, Shuxuening Injection significantly reduces angina attack frequency and duration in unstable angina patients, with effects sustained for 20 days post-treatment and a good safety profile.	The study’s limitations include a small and homogeneous sample, a short observation period preventing long-term assessment, and insufficient attention to serious outcomes.	Very low
Shexiang Baoxin Pill (57)	Stable coronary heart disease	RCT ChiCTR registration number: ChiCTRTRC-12003513	For stable coronary heart disease patients under 65 kg, the addition of Musk Heart-Protecting Pills to conventional therapy significantly reduces the risk of major cardiovascular events with good safety.	The study’s limitations include potential analytical biases, a short follow-up period obscuring long-term effects, and a small sample size that may have failed to detect significant outcomes.	Very low

These evidence-based medical studies have jointly confirmed the effectiveness of “food and medicine sharing the same origin” in the prevention and treatment of CVDs from both macroscopic levels such as epidemiological investigations and clinical efficacy statistics, and microscopic levels such as cells, molecules and biochemical pathways. Furthermore, they provide a scientific basis for further exploration of MFH’s potential role in CVDs management, while acknowledging that additional clinical validation is required.

The existing clinical evidence exhibits several common limitations. First, participant representation is narrow, as most studies enroll exclusively Chinese populations, which limits the generalizability of findings to other countries, racial groups, or broader global populations. Second, follow-up procedures are suboptimal; some trials have short observation periods, which precludes a reliable assessment of long-term efficacy, safety, and effects on critical outcomes such as myocardial infarction and survival. Additionally, high dropout rates due to external disruptions, such as the pandemic (e.g., a 63% attrition rate in the Tongxinluo Capsule study), compromise data integrity, even when mitigation strategies are applied. Third, the sample sizes in some studies are relatively small (e.g., *n* = 78 in the Yangxin Decoction trial and the Shuxuening study), which increases the risk of type II errors and limits the capacity to detect statistically significant subgroup-specific treatment effects. This limitation is further exacerbated by substantial rates of loss to follow-up (e.g., 13.3%–15.2% in the Shensong Yangxin study), which diminish statistical power and compromise the precision and reliability of the study outcomes. Fourth, efficacy assessments are incomplete; several studies fail to monitor established biomarkers of cardiovascular risk and disease progression, such as mean platelet volume in the Yangxin Decoction trial, or inflammatory markers and lipid profiles in the TXS Capsule trial. Additionally, some trials rely solely on non-specific measures (e.g., exclusive use of SF-36 in the Shensong Yangxin study) or omit monitoring for clinically significant endpoints (e.g., failure to assess myocardial infarction risk in unstable angina patients within the Shuxuening trial), thereby limiting the clinical interpretability of the results. Fifth, methodological limitations are evident across multiple studies, including the absence of randomization and blinding (e.g., syndrome differentiation-based CPM studies), the presence of uncontrolled confounding variables (e.g., variability in Acute Coronary Syndrome (ACS) subtypes), and reliance on *post hoc* subgroup analyses (e.g., MUSKARDIA-related publications), all of which introduce inherent biases and reduce internal validity. In addition, some studies did not clarify whether interventions were adjunctive or substitutive to standard therapies and exhibited measurement biases, including intermittent Atrial Fibrillation (AF) monitoring, single-time-point testosterone tests, and high thromboxane A2 variability, potentially affecting risk classification.

## 4 Strategic applications and practical recommendations for the integration of medicinal and food substances

Based on the aforementioned clinical evidence and relevant literature, we propose the following application strategies and practical recommendations for the use of MFH substances in the prevention and treatment of CVDs (Table 1).

## 5 Application strategies and practical recommendations for MFH substances

Integrating findings from prior evidence-based reviews on the adjunctive use of MFH substances in heart disease with a critical appraisal of existing research limitations—particularly regarding the representativeness of subject populations, follow-up duration, and sample size—suggests that the strategy of “Syndrome Differentiation and Diet Therapy combined with Personalized Protocols” should serve as a foundational approach for the application of MFH substances in the prevention and treatment of CVD. The implementation of this strategy must be meticulously tailored to individual patient characteristics, including physiological constitution, underlying clinical conditions, and both Western medical and TCM syndrome differentiation.

According to a comprehensive review by Yin et al., MFH substances exert multi-target cardioprotective effects against chronic heart failure (CHF) through mechanisms including oxidative stress reduction, inflammation suppression, apoptosis inhibition, and energy metabolism regulation (54). Key active components such as astragaloside IV, puerarin, curcumin, and Lycium barbarum polysaccharides are derived from common food ingredients, supporting their long-term safety and suitability as adjunctive therapies or functional foods for CHF management (54). Jin et al. developed a seven-herb MFH formula for microvascular angina through data mining and validated its multi-target mechanisms via network pharmacology and molecular docking (17). This study demonstrates that MFH formulas can be designed as safe, food-derived interventions for chronic cardiovascular conditions, thereby supporting their practical application as adjunctive therapies or functional foods in daily disease management.

Considering that different heart diseases—including heart failure, atrial fibrillation, coronary heart disease, and others—have distinct pathophysiological mechanisms and therapeutic requirements, the following clinical examples further illustrate the application of this strategy. For instance, Qiliqiangxin Capsules may serve as an adjunct to standard Western therapy for patients with heart failure with reduced ejection fraction (55). Shensongyangxin Capsules may be recommended as a 12-month adjunctive treatment following catheter ablation in patients with persistent atrial fibrillation (56). Furthermore, individual patient characteristics must be fully integrated into the decision-making process. For example, the adjunctive use of Shexiang Baoxin Pill combined with conventional therapy demonstrates a more pronounced reduction in cardiovascular event risk specifically in patients with stable coronary artery disease weighing less than 65 kg, highlighting the importance of incorporating body weight as a stratification factor in personalized treatment protocols (57).

Integrating the patient’s ongoing conventional treatment regimen is crucial to prevent potential antagonistic effects between TCM and Western medicine. This coordinated approach not only compensates for the limitations of monotherapy in improving symptoms and enhancing quality of life but also minimizes the risk of adverse interventions due to the favorable safety profile of MFH substances. The formulation and continuous adjustment of the entire protocol should be conducted under the professional supervision of a physician or clinical nutritionist, with regular monitoring of treatment responses and clinical changes, thereby achieving the goal of enhancing efficacy as an adjunct while ensuring safety. This provides a more targeted and safer pathway for translating MFH substances from theoretical research into clinical practice.

## 6 Integrated traditional Chinese and western medicine therapy and comprehensive prevention strategies

CVDs, such as hypertension, are highly prevalent chronic conditions worldwide, necessitating multi-dimensional strategies that combine drug therapy, lifestyle interventions, and nutritional regulation for effective prevention and treatment (58, 59). Recent research has increasingly focused on MFH resources, which possess both “edible safety” and “medicinal activity,” as potential adjuvant interventions for CVD. The active components of MFH regulate pathological processes associated with these diseases through multiple targets and pathways, offering novel supplementary approaches for clinical prevention and treatment. Furthermore, related studies have delineated the application boundaries and principles for their combined use in interventions. In addition to the active components found in traditional formulas, numerous studies have confirmed that MFH ingredients can provide preventive effects when consumed regularly, aiding in the prevention of conditions such as hypertension and hyperlipidemia (60–64).

A substantial body of evidence indicates that various MFH substances, due to their well-documented therapeutic potential for diseases, also hold significant commercial value for development into functional foods or as adjuvant therapeutic agents. This provides strong theoretical support for their industrial translation (65–69). It is important to emphasize that MFH cannot replace standardized medical therapies such as antihypertensive medications and statins, nor can it serve as a substitute for essential lifestyle interventions, including aerobic exercise, smoking cessation, and moderation of alcohol consumption. Rather, MFH should be integrated with conventional Western medicine under the guidance of a cardiovascular specialist or clinical nutritionist. For example, a hypertensive patient who is regularly taking antihypertensive medications, such as amlodipine, may appropriately consume MFH-based beverages, such as kudzu root (Pueraria lobata) tea or hawthorn (Crataegus pinnatifida) juice, as advised, thereby achieving a synergistic “drug plus nutrition” intervention to support dietary management in blood pressure regulation. Furthermore, in the context of daily health maintenance, increasing physical activity remains equally critical for the adjunctive CVD. Exercise can simultaneously improve depression and CVD through mechanisms like anti-inflammation and mitochondrial function enhancement (59). The “MFH + exercise” comprehensive intervention scheme thus holds practical value: MFH exerts anti-inflammatory, antioxidant, and gut microbiota-regulating effects, while exercise optimizes autonomic nervous system balance and vascular endothelial function. The two complement each other to improve cardiovascular parameters and alleviate depressive symptoms, thereby enhancing the feasibility of CVD prevention. Evidence suggests that gentle exercise modalities, such as Tai Chi, can help reduce cardiovascular risk in individuals with prehypertension (70, 71). Further research supports the notion that traditional Chinese exercises can significantly enhance physiological indicators, such as blood pressure and heart rate, and biochemical markers, including blood lipids and blood sugar levels, in patients with CVD. Additionally, these exercises improve physical function and quality of life while alleviating psychological issues, such as depression. This evidence provides patients with exercise intervention options that are more consistent with traditional health preservation concepts (72).

In summary, MFH resources offer a supplementary approach to CVD prevention and treatment through a combination of dietary assistance and lifestyle optimization. This dual strategy not only expands the methods available for clinical intervention but also addresses the public’s demand for low-toxicity and convenient treatment options. However, it is essential to clarify its role as an “adjuvant” therapy, ensuring that it is used in conjunction with standardized treatments and guided by professional advice. This approach will enhance its effectiveness in the prevention and management of CVD, ultimately providing more comprehensive protection for patient health.

## 7 Optimizing the application of MFH substances through modern technology

While traditional MFH ingredients possess the potential to aid in the regulation of cardiovascular function, their clinical application has historically encountered challenges due to factors such as low absorption efficiency and a lack of personalized treatment protocols. Modern technologies can be leveraged to enhance the applicability of MFH substances in the management of CVDs. For instance, fermenting HT into an oral liquid formulation can improve the bioavailability of active components, thereby facilitating administration for elderly patients with cardiovascular conditions. Extracting crataegic acid from hawthorn (Crataegus pinnatifida) allows for the production of standardized capsules, which can serve as adjuvant therapy for patients with coronary heart disease. Furthermore, developing personalized dietary plans that incorporate Poria cocos, Coix lacryma-jobi (Job’s tears), and Dioscorea opposita (Chinese yam) can benefit patients with hyperlipidemia and concurrent gut microbiota dysbiosis, all guided by metabolomic profiling of lipid markers and gut microbiota analysis. Polysaccharides from these MFH sources exert anti-obesity effects through the gut-liver axis, encompassing the modulation of fat metabolism, appetite control, intestinal microbiota, and systemic inflammation (73), while hawthorn exhibits antihypertensive, lipid-lowering, and antioxidant properties that help prevent atherosclerosis (74). These technology-driven strategies enable MFH substances to assume a more precise and effective role as adjunctive interventions in the prevention and treatment of CVDs.

In addition to advancements in ingredient processing and protocol customization, contemporary technology facilitates the comprehensive integration of MFH with the diagnosis and treatment of CVDs. Some studies have employed artificial intelligence to enhance the quality assessment of MFH ingredients, thereby ensuring their safety and efficacy. For instance, modeling of moisture content using near-infrared spectroscopy allows for the rapid determination of water content in MFH substances (75). Recent studies indicate that the integration of artificial intelligence with both Chinese and Western medicine for the diagnosis and treatment of CVDs has emerged as a significant avenue for industrial innovation (76). Combined with multimodal imaging and biomarker-based precise diagnostic technologies (77), AI can further integrate patients’ genetic polymorphisms (such as CYP2C19), providing a more accurate basis for the personalized compatibility of MFH. For instance, it can screen MFH components with synergistic antiplatelet effects for CVD patients who have an inadequate response to antiplatelet therapy. For example, in the realm of auxiliary diagnosis and clinical decision support, machine learning algorithms can be employed to develop non-invasive predictive models for coronary heart disease based on echocardiographic features and clinical characteristics. These algorithms can also be used to identify coronary artery lesions through radial artery pulse wave analysis, enabling the differentiation of patients with stable coronary heart disease. Furthermore, weakly supervised deep learning techniques can assist in TCM syndrome differentiation by identifying the ‘teeth-marked tongue’ phenomenon. Additionally, multi-graph convolutional networks and Transformer architecture systems can enhance the accuracy of TCM prescription recommendations and facilitate the provision of integrated prescription suggestions that combine both Chinese and Western medicinal approaches (78).

The global promotion of the MFH concept not only offers innovative perspectives for the prevention and treatment of CVD but also revitalizes the development of the global functional food and health industry by incorporating traditional wisdom. The synergistic integration of MFH with modern advanced technologies preserves the benefits of being “natural and low-toxicity” while harnessing technological advancements to achieve efficient and precise enhancements. In the future, as technology matures and clinical evidence accumulates, MFH resources are anticipated to play a more significant adjuvant role in the global prevention and treatment of CVD. This will provide patients with options that balance health and quality of life, while also fostering a deeper integration of traditional health preservation practices with modern medicine, thereby contributing to global health initiatives.

## 8 Challenges and future prospects of medicine and food homology (MFH) in clinical treatment

Although MFH substances have shown potential in CVD prevention and adjunctive management, several limitations remain in their clinical translation and application. First, most clinical studies are single-center designed with small-sample size and short follow-up durations, resulting in insufficient evidence regarding major endpoints such as cardiovascular mortality, myocardial infarction, and stroke. In addition, the disparity between preclinical investigations and human studies remains substantial, which limits the strength of current clinical recommendations. Second, MFH substances comprise various bioactive components, including polysaccharides, polyphenols, and saponins, whose synergistic and antagonistic effects remain unclear. Furthermore, current research on the metabolic pathways of TCM is inadequate, with a notable lack of data regarding intestinal microbial metabolism, bioavailability, and pharmacokinetics, which collectively obscure the identification of key bioactive constituents and their therapeutic targets. The variations in the planting environment and processing technologies may also lead to inconsistencies in composition and quality of MFH substances. The concomitant use of TCM with Western medications, such as warfarin and Angelica sinensis, may elevate the risk of bleeding or potential drug interactions. Additionally, the safety profile of certain MFH substances, such as Rehmannia glutinosa and Gastrodin elata, requires continued pharmacovigilance, although current dietary consumption remains within safe ranges and necessitates systematic evaluation. Collectively, these issues highlight critical gaps in current quality assurance and safety surveillance systems for MFH substances.

In addition, existing evidence indicates that subclinical myocardial dysfunction is highly prevalent in individuals with metabolic syndrome, prediabetes, and metabolic dysfunction-associated steatotic liver disease (MASLD). Importantly, speckle-tracking echocardiography (STE) enables its early detection through the identification of abnormal myocardial strain parameters (79). For instance, Li et al. demonstrated that syringin, a natural phenolic compound, significantly improved global longitudinal strain (GLS) and global circumferential strain (GCS) in mice with myocardial injury, further supporting the utility of STE in both early identification and therapeutic evaluation (80). Multiple metabolic disturbances, including chronic inflammation (81), insulin resistance, oxidative stress, endothelial dysfunction, ectopic fat deposition, and gut microbiota dysbiosis (82), interact with each other to jointly drive subclinical myocardial injury and adverse ventricular remodeling. Notably, subclinical myocardial injury is associated with a more than threefold increased risk of heart failure, which is further amplified to more than fourfold when prediabetes is present, highlighting the critical “amplification effect” of metabolic abnormalities on the progression of myocardial injury (83). Interestingly, these metabolic disturbances are core regulatory targets of MFH substances. These substances exert synergistic protective effects through multiple pathways, including anti-inflammation, antioxidant activity, improvement of insulin sensitivity, regulation of lipid metabolism, protection of vascular endothelium, and modulation of gut microbiota, thereby both blocking the sustained damage caused by metabolic disorders and directly improving myocardial structure and function at the subclinical stage. Taken together, MFH substances may represent feasible long-term early intervention strategies for MASLD-related subclinical myocardial dysfunction, although confirmatory studies are needed .

## 9 Conclusion

In summary, the present review systematically summarizes the multi-dimensional cardiovascular protective effects and underlying molecular mechanisms of MFH substances in the prevention and auxiliary clinical management of CVD. The protective properties of MFH substances can be generalized into four interrelated and complementary core modules: vascular antioxidant and anti-inflammatory defense, myocardial function protection and metabolic regulation, gut-heart axis homeostasis modulation, and comprehensive intervention of cardiovascular metabolic risk factors. Together, these domains establish a systematic theoretical framework for MFH substances to improve cardiovascular health.

Mechanistically, MFH-derived diverse bioactive compounds exert multi-target and multi-pathway protective effects on the cardiovascular system. These active ingredients effectively regulate core signaling cascades associated with oxidation and inflammation, significantly reducing reactive oxygen species accumulation and pro-inflammatory cytokine release, and thereby protecting vascular endothelial structure and functional integrity to prevent vascular pathological remodeling. Moreover, MFH bioactives optimize myocardial glycolipid metabolism and mitochondrial energy homeostasis, suppress excessive cardiomyocyte apoptosis, and stabilize cardiac systolic and diastolic functions, which contribute to the mitigation of myocardial damage. Notably, MFH polysaccharides and other functional components can reshape intestinal microbiota composition, elevate beneficial short-chain fatty acid production, reduce harmful metabolic toxins, and maintain intestinal barrier integrity. Through the gut-heart axis crosstalk, these substances alleviate systemic metabolic disorders and chronic inflammation, realizing indirect cardiovascular protective effects. In addition, MFH substances exhibit favorable regulatory effects on cardiovascular risk factors by inhibiting lipid absorption and improving obesity-related dyslipidemia, thus intervening in the progression of atherosclerotic CVD at the metabolic source.

Existing preliminary clinical observations have confirmed that MFH-based compound preparations can serve as safe and effective adjuvant therapies for multiple common CVDs. Combined with conventional Western medicine treatments, these formulations can efficiently relieve clinical discomforts, improve patients’ quality of life, and lower the incidence of adverse cardiovascular events with reliable safety performance. Nevertheless, current clinical research on MFH substances for CVD treatment still possesses evident limitations, including small sample sizes, insufficient long-term follow-up data, narrow population adaptability, and non-uniform experimental protocols. These deficiencies greatly restrict the clinical popularization, standardized application, and systematic evaluation of MFH therapeutic efficacy. In terms of application strategies, the combination of TCM syndrome differentiation and individualized dietary intervention provides a scientific and feasible guiding principle for the clinical auxiliary application of MFH substances, which can maximize their therapeutic advantages and ensure medication safety on the premise of standardized conventional treatment.

## 10 Future direction

Future research on medicine-food homology (MFH) in cardiovascular disease (CVD) management should focus on optimized study design, standardized quality control, precision stratified intervention, and technology-driven translational innovation, to accelerate the transition of MFH from empirical application to evidence-based, standardized, and personalized clinical practice.

First, upgrade clinical research design to generate high-level evidence. Large-scale, multi-center, long-term follow-up randomized controlled trials (RCTs) should be prioritized, enrolling multi-ethnic and multi-regional populations to improve global generalizability. Trial outcomes should incorporate hard clinical endpoints including cardiovascular mortality, myocardial infarction, and stroke, together with structured cardiovascular biomarkers and imaging indicators such as speckle-tracking echocardiography-derived myocardial strain parameters. Moreover, standardized reporting of systematic reviews and meta-analyses should adopt the AMSTAR-2 framework to strictly control selection bias, publication bias, and confounding bias, thereby elevating the credibility of cumulative clinical evidence. Embedded stepped-wedge trials combined with real-world evidence cohorts are encouraged to better reflect routine clinical scenarios and support practical guideline formulation.

Second, establish whole-process standardized quality control system for MFH substances. Future work should unify protocols for germplasm conservation, ecological cultivation, harvesting season, post-harvest processing, and extraction technology. The Quality by Design (QbD) framework should be introduced to define critical material attributes (CMA) and critical process parameters (CPP), constructing a full-chain quality management covering cultivation, processing, component extraction, and formulation preparation. Multi-batch and multi-regional comparative studies are needed to clarify the influence of geographical and technological heterogeneity on phytochemical composition and bioactivity, ensuring the consistency, comparability, and reproducibility of preclinical and clinical research results. International harmonization of regulatory and technical standards for MFH botanical materials should also be promoted to facilitate global academic and clinical recognition.

Third, develop precision stratification and individualized application strategies. Future clinical implementation should build phenotype-based precise grouping frameworks, stratifying patients according to metabolic subtypes, gut microbiota enterotypes, disease stages (subclinical phase, acute phase, rehabilitation phase), and comorbidity profiles. Clear application boundaries should be defined for MFH adjuvant intervention in combination with conventional Western therapies (antiplatelet agents, statins, ACEI/ARB), forming evidence-based recommendations for synergistic supplementation rather than simple replacement. Unified specifications of formulation type, standardized dosage, administration route, and intervention course should be established for different CVD phenotypes. Particularly for high-risk populations such as MASLD, prediabetes, and subclinical myocardial dysfunction, long-term early MFH dietary intervention regimens should be formulated under TCM syndrome differentiation, weight stratification, and constitutional classification to achieve individualized cardiovascular protection. Meanwhile, herb–drug interaction risks between MFH ingredients and anticoagulant/antiplatelet drugs should be systematically summarized to formulate safe medication guidance for clinical co-administration.

Fourth, integrate multi-omics and artificial intelligence technologies to deepen mechanism and translational research. Multi-omics strategies incorporating network pharmacology, metabolomics, microbiome and toxicogenomics should be combined to systematically decipher the synergistic and antagonistic relationships among MFH bioactive components, core targets, and downstream signaling pathways. Artificial intelligence and machine learning algorithms can be applied to rapid ingredient quality evaluation, active component screening, off-target toxicity prediction, and herb–drug interaction risk early warning. Combined with multimodal cardiovascular imaging, genetic polymorphism detection (e.g., CYP450 subtypes), and gut microbiota profiling, AI-assisted models can provide precise compatibility suggestions for personalized MFH prescription and dietary intervention. Furthermore, an open-access MFH component database and clinical trial registration platform should be constructed to realize data sharing, evidence integration, and resource reuse.

Fifth, explore digital health model and pharmacoeconomic evaluation. Future studies should integrate wearable monitoring, mobile health management, and dynamic lifestyle intervention to build an innovative “MFH + digital health” model, enabling real-time monitoring, feedback adjustment, and long-term personalized dietary intervention for CVD high-risk groups. In parallel, large-sample real-world studies should be conducted to perform cost-effectiveness and cost-utility analyses, quantifying the value of MFH adjuvant therapy in reducing cardiovascular event recurrence and medical resource consumption. Relevant pharmacoeconomic evidence can further support health technology assessment and clinical popularization of MFH-based preventive and rehabilitative strategies.

Collectively, through high-evidence clinical design, standardized quality assurance, precision stratified application, and multi-technology cross-integration, MFH substances can be further developed into safe, effective, and cost-efficient natural adjuvant strategies for the comprehensive prevention and long-term management of cardiovascular diseases.
