# Supplementary material for: Medicine-food homology substances in cardiovascular disease prevention and management: from mechanisms to clinical evidence and future translation
Source: Front Nutr. 2026 Jun 25;13:1825606. doi: 10.3389/fnut.2026.1825606 (PMC13345857; doi:10.3389/fnut.2026.1825606)
Supplement: Supplementary file 1 [file Table_1.DOCX]

Supplementary Material

Medicine-food homology substances in cardiovascular disease prevention and management: from mechanisms to clinical evidence and future translation

# Supplementary Tables

**Table A1: Overview of Relevant Research on Various Types of Medicine-Food Homology Active Substances in Intervening Cardiovascular Diseases.**

| Category | Classification | Chinese Herbal Medicine / Extract / Monomer Name | Primary Active Ingredient | Effective biological activity | Cardiovascular diseases involved | Research Findings | Clinical Application Scenario | Source of Documentation |
| --- | --- | --- | --- | --- | --- | --- | --- | --- |
| Triterpenoid saponins | MFH monomer | Astragalosides | Astragaloside IV, cycloartane-type triterpene saponins (e.g., Astraoleanosides E-P) | Antioxidant, anti-inflammatory, neuroprotective, β-glucuronidase inhibitor, tumor cell proliferation and migration inhibitor, vascular endothelial function protector | Heart failure, myocardial ischemia-reperfusion injury, atherosclerosis | Reduces oxidative stress and glial cell activation in the hippocampus, preventing inflammatory cell infiltration; improves neurological function scores and reduces infarct volume in rats with focal cerebral ischemia-reperfusion injury;  Inhibits the Vav3/Rac1/MAPK pathway, lowers MMP expression, and suppresses tumor angiogenesis;  3. Modulates the PI3K/Akt/NF-κB pathway to protect vascular endothelial function. | Adjuvant intervention for heart failure, myocardial ischemia-reperfusion injury, and atherosclerosis | (1-7) |
| Polysaccharides | MFH monomer | Astragalus Polysaccharides | Linear glucose units (linked 1→4), oligosaccharides (e.g., octosaccharides) | Antioxidant, anti-inflammatory, immunomodulatory, improves insulin resistance, protects pancreatic β-cells, inhibits tumor cell apoptosis | Diabetes, diabetic cardiovascular complications, viral myocarditis | Medium molecular weight (4.72 kDa) APS exhibits the strongest antioxidant activity, enhancing SOD activity and inhibiting MDA release;  Activates NF-κB and TLR4 pathways, promoting macrophage phagocytosis and cytokine secretion;  3. Modulates insulin signaling pathways, enhances IRS-1 tyrosine phosphorylation and GLUT4 translocation, improving insulin resistance;  4. Inhibits LPS-induced inflammatory cytokine release in RAW264.7 cells, protecting myocardial tissue in CVB3-induced viral myocarditis mice. | Dietary adjuvant intervention for diabetes, diabetic cardiovascular complications, and viral myocarditis | (6, 8-13) |
| Flavonoids | MFH monomer | Total Flavonoids of Astragalus | Isoflavones, flavonols, dihydroflavones, etc. (such as genistein, genistein isoflavone, quercetin, etc.) | Antioxidant, anti-inflammatory, immune cell function regulation, α-glucosidase inhibition, tyrosinase inhibition | Heart failure, diabetic cardiovascular complications | Scavenges free radicals and terminates free radical chain reactions; chelates metal ions such as Fe²⁺ and Cu²⁺ to inhibit free radical generation;  Enhances the activity of antioxidant enzymes including SOD and GSH-Px;  Regulates inflammatory pathways such as NF-κB and MAPK to reduce the release of inflammatory mediators like TNF-α and IL-6;  4. Improves high-glucose-induced inflammatory damage in HK-2 cells. | Adjuvant nutritional intervention for heart failure and diabetic cardiovascular complications | (6, 7, 14-20) |
| Medicinal Foods (Fruit Category) | MFH natural material | Tsaoko Fructus | Polyphenol extracts, essential oils | Regulate CYP7A1 expression, modulate gut microbiota, and promote cholesterol conversion | Atherosclerosis (related to hyperlipidemia) | Alter the abundance of gut microbiota associated with CYP7A1, including Allobaculum, Desulfovibrio, and Ruminococcus_2;  Upregulate CYP7A1 mRNA and protein expression, promoting cholesterol conversion to bile acids and excretion to exert cholesterol-lowering effects;  3. Intervene in Syrian hamsters to improve hyperlipidemia through gut microbiota regulation, thereby indirectly mitigating atherosclerosis risk. | Dietary intervention for hyperlipidemia and related atherosclerosis | (21) |
| Medicinal and edible plants | Single medicinal food homologous plant | Barley leaf (*Hordeum vulgare L.*) | Plant polyphenols, dietary fiber, natural antioxidants | Intestinal flora regulation, intestinal barrier protection, anti-inflammation, myocardial protection | Myocardial infarction; ischemic cardiovascular disease | Barley leaf remodels gut microbiota and enriches *Lachnospiraceae*. Its derived extracellular vesicles regulate ERα‑Slc6a14‑Hippo axis to improve gut-heart axis disorder and relieve post-infarction cardiac remodeling. | Adjuvant nutritional intervention for coronary heart disease and myocardial infarction; daily dietary regulation for high-risk cardiovascular populations; intestinal microecology improvement for cardiac patients. | (22) |
| Medicinal and edible plants | Compound herbal preparation | *Hippophae rhamnoides L.* & *Silybum marianum* (L.) Gaertn. solid beverage | Flavonoids, silymarin, polyphenols | Lipid-lowering, antioxidant, hepatoprotective, anti-obesity, lipid metabolism regulation | Hyperlipidemia; cardiovascular metabolic diseases | This compound beverage contains 89 bioactive components. It markedly reduces body weight, lipid deposition and liver injury in high-fat diet rats, and modulates cholesterol synthesis, lipogenesis and fatty acid β‑oxidation via multi-omics regulation. | Serving as an auxiliary daily intervention for hyperlipidemia; preventing cardiovascular risks induced by abnormal lipid metabolism; liver protection for metabolic population (preclinical stage). | (23) |
| Medicinal and edible plant | Single medicinal and edible homologous plant (polysaccharide extract) | *Allium Macrostemon Bge.* (AMB), crude polysaccharides of AMB (AMBP), homogeneous polysaccharide AMBP80-1a | Macrostemon bulb polysaccharide (main component: AMBP80-1a, agavin-type fructan) | Anti-atherosclerosis, reduce lipid accumulation, lower cholesterol, inhibit foam cell formation | Atherosclerosis | *Allium Macrostemon Bge.* is rich in polysaccharides. AMBP exerts anti-atherosclerotic effects in vivo and in vitro. The purified homogeneous polysaccharide AMBP80-1a has a yield of 11.1% and a molecular weight of 10.01 kDa. It can reduce lipid and cholesterol accumulation in ox-LDL-induced THP-1 foam cells. | It can be used as a functional ingredient for atherosclerosis prevention and dietary conditioning for cardiovascular high-risk groups, serving as auxiliary intervention for atherosclerosis (still in basic research and pending clinical verification) | (24) |
| Medicinal and edible plant (TCM) | Compound TCM preparation | Dangshen Formula Shengmai-Yin (DS-SMY) | Lobetyolin, schisandrin, and other active components (identified by HPLC) | Anti-atherosclerosis, inhibiting macrophage M1 polarization, relieving intestinal inflammation, repairing intestinal tight junctions, regulating gut microbiota, suppressing proinflammatory cytokine expression, enhancing macrophage efferocytosis | Atherosclerosis (AS), dyslipidemia caused by AS, intestinal inflammation associated with AS | 1. DS-SMY has a therapeutic effect on atherosclerosis in high-fat diet-induced ApoE⁻/⁻ mice, which is comparable to atorvastatin; 2. It can inhibit macrophage M1 polarization, alleviate intestinal inflammation, repair colon tight junctions, and reshape gut microbiota; 3. Its active components (lobetyolin, schisandrin) can suppress proinflammatory cytokine expression and enhance macrophage efferocytosis; 4. The therapeutic effect of DS-SMY is related to the gut microbiota-dependent mechanism, and butyric acid (a metabolite of gut microbiota) can synergistically enhance its efficacy.  5.Atorvastatin was used as the positive control drug. The therapeutic effect of DS-SMY on atherosclerosis is comparable to that of Atorvastatin; DS-SMY is superior to Atorvastatin in repairing intestinal barrier and regulating gut microbiota, while Atorvastatin has advantages in rapid lipid-lowering and inhibiting vascular lipid deposition. | It can be used as an auxiliary intervention for atherosclerosis, helping to alleviate lipid deposition and vascular inflammation; it can also assist in regulating intestinal microecology and reducing the risk of cardiovascular diseases caused by atherosclerosis (pending clinical verification). | (25) |
| Single-component compounds (polysaccharides) | MFH monomer | Goji Polysaccharides | Goji Polysaccharides | Anti-inflammatory, antioxidant, anti-apoptotic, and modulates gut microbiota | Heart failure, myocardial ischemia-reperfusion injury, cardiac hypertrophy | Reduced levels of inflammatory cytokines (such as IL-6, TNF-α) and plasma lipid peroxidation in a rat model of stress-induced heart failure;  Protect rats and cardiomyocytes from ischemia-reperfusion injury by activating Nrf2 and inhibiting autophagy;  Alleviate cardiac hypertrophy in streptozotocin-induced diabetic rats by suppressing calpain-1 expression and NF-κB activation;  4. Mitigate myocardial injury in high-fat diet-fed mice by modulating the gut microbiome and fecal metabolome. | Adjuvant intervention and rehabilitation support for heart failure, myocardial ischemia-reperfusion injury, and cardiac hypertrophy | (26-29) |

**Table A2: List of medicine-food homology (MFH) substances, bioactive components and related compound preparations**

| MFH Substance (Common Name & Scientific Name) | Core Bioactive Components | Related Compound Preparations |
| --- | --- | --- |
| Astragalus (Mongolian Milkvetch, Astragalus membranaceus) | Astragaloside IV, astragalus polysaccharide, triterpenoid saponins, isoflavonoids | Seven-herb MFH formula, Yangxin Recipe, Tongxinluo Capsules, Qiliqiangxin Capsules |
| Kudzu Root (Pueraria lobata / Pueraria montana var. thomsonii) | Puerarin, low-molecular-weight polysaccharide, isoflavonoids | Seven-herb MFH formula |
| Licorice (Glycyrrhiza uralensis) | Isoliquiritigenin, soyasaponin, triterpenoid saponins, flavonoids | Seven-herb MFH formula |
| Soybean (Glycine max) | Genistein, soyasaponin, isoflavonoids, saponins | — |
| Longstamen Onion Bulb (Allii Macrostemonis Bulbus) | Allium macrostemon saponins, steroidal saponins | Seven-herb MFH formula |
| Danshen (Salvia miltiorrhiza) | Salvia miltiorrhiza hydrophilic extract, salvianolic acids, tanshinones | Yangxin Recipe, Danhong Injection |
| Mulberry Leaf (Morus alba) | Mulberry leaf polysaccharide, flavonoids, alkaloids | — |
| Grosvenor Momordica (Siraitia grosvenorii) | Siraitia grosvenorii polysaccharide, triterpenoids | — |
| Chinese Angelica (Angelica sinensis) | Angelica polysaccharide, ferulic acid, flavonoids | Seven-herb MFH formula, Yangxin Recipe |
| Solomon's Seal (Polygonatum sibiricum) | Polygonatum polysaccharide, steroidal saponins, flavonoids | — |
| Goji Berry (Lycium barbarum / Lycium chinensis) | Lycium barbarum polysaccharide, carotenoids, flavonoids | — |
| Raspberry (Rubus idaeus) | Raspberry polysaccharide, ellagic acid, flavonoids | — |
| Reishi Mushroom (Ganoderma lucidum) | Ganoderma lucidum polysaccharide, triterpenoids | — |
| Cape Jasmine (Gardenia jasminoides) | Gardenia polysaccharide, geniposide, iridoids | — |
| Hawthorn (Crataegus pinnatifida / Crataegus oxyacantha) | Crataegic acid, vitexin-2″-O-rhamnoside, flavonoids, triterpenoids | — |
| Turmeric (Curcuma longa) | Curcumin, curcuminoids, phenolic acids | — |
| Lotus Leaf (Nelumbo nucifera) | Nuciferine, flavonoids, alkaloids | — |
| Sea Buckthorn (Hippophae rhamnoides) | Sea buckthorn flavonoids, polyphenols, vitamins | — |
| Codonopsis (Codonopsis pilosula) | Codonopsis polysaccharide, triterpenoids, alkaloids | Seven-herb MFH formula |
| Balloon Flower Root (Platycodon grandiflorum) | Platycodin, polysaccharides, saponins | Seven-herb MFH formula |
| Peach Kernel (Prunus persica) | Amygdalin, flavonoids, phenolic acids | Seven-herb MFH formula |
| Ginseng (Panax ginseng) | Ginsenosides, polysaccharides, triterpenoids | Tongxinluo Capsules |
| White Peony Root (Paeonia lactiflora) | Paeoniflorin, polysaccharides, flavonoids | Tongxinluo Capsules |
| Safflower (Carthamus tinctorius) | Hydroxysafflor yellow A, flavonoids, alkaloids | Danhong Injection |
| Ginkgo Leaf (Ginkgo biloba) | Ginkgolide B, flavonoid glycosides, terpenoids | Shuxuening Injection |

**Common Adverse Reactions**

# Gastrointestinal reactions: Abdominal distension, diarrhea, nausea, acid reflux (most common, incidence < 5%, mostly transient).

# Allergic reactions: Skin rash, pruritus (very rare, mostly in flavonoids/phenolic acids).

# Metabolism-related: No hypoglycemia, myalgia, or liver enzyme elevation (superior to conventional chemical drugs).

# Long-term safety: All MFH monomers show no severe organ toxicity and are suitable for long-term dietary or adjuvant intervention.

# Drug interactions: Monitoring for bleeding risk is recommended when combined with anticoagulants (warfarin) or antiplatelet agents (theoretical suggestion, rare in clinical practice).

**Evidence Grading**

All included studies were preclinical in vivo and in vitro experiments. According to the GRADE approach, the overall certainty of evidence was rated as very low due to high indirectness and lack of clinical data.

Given that the current evidence for medicine-food homology (MFH) substances in cardiovascular protection is rated as very low certainty using the GRADE approach, primarily derived from preclinical in vitro and animal studies with high indirectness, future research priorities should focus on translational and clinical validation.

First, well-designed randomized controlled trials (RCTs) in humans are urgently needed to verify the efficacy, optimal dosage, and long-term safety of MFH products in real-world cardiovascular populations. Second, large-scale prospective cohort studies should be conducted to evaluate real-world effectiveness, adverse reactions, and potential drug–herb interactions. Third, standardized extraction, quality control, and formulation development of active ingredients (such as polysaccharides, flavonoids, and saponins) are required to improve clinical applicability.

In addition, multi-omics and mechanistic studies should be further integrated to clarify the tissue-specific targets and signaling pathways of MFH substances. Ultimately, high-quality clinical evidence will help upgrade the GRADE level of evidence and support the rational clinical translation of MFH products for the prevention and adjuvant treatment of cardiovascular diseases.

**Reference**

1. Costa IM, Lima FO, Fernandes LC, Norrara B, Neta FI, Alves RD, et al. Astragaloside IV supplementation promotes a neuroprotective effect in experimental models of neurological disorders: a systematic review. Current neuropharmacology. 2019;17(7):648-65.

2. Yang J, Li J, Lu J, Zhang Y, Zhu Z, Wan H. Synergistic protective effect of astragaloside IV–tetramethylpyrazine against cerebral ischemic-reperfusion injury induced by transient focal ischemia. Journal of Ethnopharmacology. 2012;140(1):64-72.

3. Jiang K, Lu Q, Li Q, Ji Y, Chen W, Xue X. Astragaloside IV inhibits breast cancer cell invasion by suppressing Vav3 mediated Rac1/MAPK signaling. International Immunopharmacology. 2017;42:195-202.

4. Leng B, Tang F, Lu M, Zhang Z, Wang H, Zhang Y. Astragaloside IV improves vascular endothelial dysfunction by inhibiting the TLR4/NF-κB signaling pathway. Life Sciences. 2018;209:111-21.

5. Zhu J, Wen K. Astragaloside IV inhibits TGF‐β1‐induced epithelial‐mesenchymal transition through inhibition of the PI3K/Akt/NF‐κB pathway in gastric cancer cells. Phytotherapy Research. 2018;32(7):1289-96.

6. Chen G, Jiang N, Zheng J, Hu H, Yang H, Lin A, et al. Structural characterization and anti-inflammatory activity of polysaccharides from Astragalus membranaceus. International Journal of Biological Macromolecules. 2023;241:124386.

7. Meng Q, Du X, Wang H, Gu H, Zhan J, Zhou Z. Astragalus polysaccharides inhibits cell growth and pro-inflammatory response in IL-1β-stimulated fibroblast-like synoviocytes by enhancement of autophagy via PI3K/AKT/mTOR inhibition. Apoptosis. 2017;22(9):1138-46.

8. Wang J-M, Sun X-Y, Ouyang J-M. Structural characterization, antioxidant activity, and biomedical application of astragalus polysaccharide degradation products. International Journal of Polymer Science. 2018;2018(1):5136185.

9. Feng S, Ding H, Liu L, Peng C, Huang Y, Zhong F, et al. Astragalus polysaccharide enhances the immune function of RAW264. 7 macrophages via the NF-κB p65/MAPK signaling pathway. Experimental and therapeutic medicine. 2021;21(1):20.

10. Liu M, Wu K, Mao X, Wu Y, Ouyang J. Astragalus polysaccharide improves insulin sensitivity in KKAy mice: Regulation of PKB/GLUT4 signaling in skeletal muscle. Journal of Ethnopharmacology. 2010;127(1):32-7.

11. Ye Y, Deng T, Wan X-Y, Ouyang J-P, Liu M, Mao X-Q. The role of quantitative changes in the epxression of insulin receptor substrate-1 and nuclear ubiquitin in abnormal glycometabolism in the livers of KKay mice and the relative therapeutic mechanisms of Astragalus polysaccharide. International journal of molecular medicine. 2014;33(2):341-50.

12. Wu Y, Ou-yang J-p, Wu K, Wang Y, Zhou Y-f, Wen C-y. Hypoglycemic effect of Astragalus polysaccharide and its effect on PTP1B. Acta Pharmacologica Sinica. 2005;26(3):345-52.

13. Liu T, Zhang M, Niu H, Liu J, Ruilian M, Wang Y, et al. Astragalus polysaccharide from Astragalus Melittin ameliorates inflammation via suppressing the activation of TLR-4/NF-κB p65 signal pathway and protects mice from CVB3-induced virus myocarditis. International Journal of Biological Macromolecules. 2019;126:179-86.

14. Lu X-Q, Qin S, Li J. Radical Scavenging Capability and Mechanism of Three Isoflavonoids Extracted from Radix Astragali: A Theoretical Study. Molecules. 2023;28(13):5039.

15. Cherrak SA, Mokhtari-Soulimane N, Berroukeche F, Bensenane B, Cherbonnel A, Merzouk H, et al. In vitro antioxidant versus metal ion chelating properties of flavonoids: A structure-activity investigation. PloS one. 2016;11(10):e0165575.

16. Hao Z, Li Z, Huo J, Li J, Liu F, Yin P. Effects of Chinese wolfberry and Astragalus extract on the antioxidant capacity of Tibetan pig liver. PloS one. 2021;16(1):e0245749.

17. Chen W, Sun Q, Ju J, Chen W, Zhao X, Zhang Y, et al. Astragalus polysaccharides inhibit oxidation in high glucose-challenged or SOD2-silenced H9C2 cells. Diabetes, metabolic syndrome and obesity: targets and therapy. 2018:673-81.

18. Wang Y, Jiang F, Cheng H, Tan X, Liu Y, Wei C, et al. Astragaloside IV Protects Against Oxidative Stress in Calf Small Intestine Epithelial Cells via NFE2L2-Antioxidant Response Element Signaling. International Journal of Molecular Sciences. 2019;20(24):6131.

19. Li K, Chen Y, Jiang R, Chen D, Wang H, Xiong W, et al. Protective effects of astragaloside IV against ovalbumin-induced allergic rhinitis are mediated by T-box protein expressed in T cells/GATA-3 and forkhead box protein 3/retinoic acid-related orphan nuclear receptor γt. Molecular medicine reports. 2017;16(2):1207-15.

20. Adesso S, Russo R, Quaroni A, Autore G, Marzocco S. Astragalus membranaceus Extract Attenuates Inflammation and Oxidative Stress in Intestinal Epithelial Cells via NF-κB Activation and Nrf2 Response. International Journal of Molecular Sciences. 2018;19(3):800.

21. Liu L, Zhao Y, Ming J, Chen J, Zhao G, Chen Z-Y, et al. Polyphenol extract and essential oil of Amomum tsao-ko equally alleviate hypercholesterolemia and modulate gut microbiota. Food & Function. 2021;12(23):12008-21.

22. Chen W, Zhao Y, Zhao Q, Zhou Y, Ma C, Dong L, et al. Lachnospiraceae-Derived Extracellular Vesicles Mediate the Cardioprotective Effects of Barley Leaf in Myocardial Infarction by Improving Intestinal Stem Cell Function. J Extracell Vesicles. 2026;15(3):e70250.

23. Bai Y, Li J, Wu X, Zhang M, Zhang Y, Chen P, et al. Mult-omics analysis reveals the lipid-lowering effects of sea buckthorn and milk thistle solid beverage in hyperlipidemic rats. Phytomedicine. 2025;144:156920.

24. Lin P, Wang Q, Wang Q, Chen J, He L, Qin Z, et al. Evaluation of the anti-atherosclerotic effect for Allium macrostemon Bge. Polysaccharides and structural characterization of its a newly active fructan. Carbohydr Polym. 2024;340:122289.

25. Li Y, Wang F, Hu M, Li Y, Xia S, Zhou Y, et al. Dangshen formula Shengmai-Yin suppresses atherosclerosis through restoring the gut microbiota and homeostatic efferocytosis. Phytomedicine. 2026;156:158210.

26. Pop C, Berce C, Ghibu S, Scurtu I, Sorițău O, Login C, et al. Effects of Lycium barbarum L. Polysaccharides on Inflammation and Oxidative Stress Markers in a Pressure Overload-Induced Heart Failure Rat Model. Molecules. 2020;25(3):466.

27. Zhang Z, Liu H, Yu B, Tao H, Li J, Wu Z, et al. Lycium barbarum polysaccharide attenuates myocardial injury in high-fat diet-fed mice through manipulating the gut microbiome and fecal metabolome. Food Research International. 2020;138:109778.

28. Pan H, Niu L, Wu Y, Chen L, Zhou X, Zhao Y. Lycium barbarum polysaccharide protects rats and cardiomyocytes against ischemia/reperfusion injury via Nrf2 activation through autophagy inhibition. Molecular medicine reports. 2021;24(5):778.

29. Liu Q, Han Q, Lu M, Wang H, Tang F. Lycium barbarum polysaccharide attenuates cardiac hypertrophy, inhibits calpain-1 expression and inhibits NF-κB activation in streptozotocin-induced diabetic rats. Experimental and therapeutic medicine. 2019;18(1):509-16.
